# Dry Eye in Colombian Tomato Farmers: An Exploratory Cross-Sectional Study of Occupational Exposure Duration

**DOI:** 10.3390/jpm16050264

**Published:** 2026-05-14

**Authors:** María Catalina Morón Barreto, José-María Sánchez-González, Diana Cristina Palencia Florez

**Affiliations:** 1 Group Interdisciplinario de Investigaciones Epidemiológicas en el Sistema Visual—GIESVI, Department of Optometry, Optometry School, Saint Thomas University, Bucaramanga 680001, Colombia; maria.moron@ustabuca.edu.co (M.C.M.B.); diana.palencia@ustabuca.edu.co (D.C.P.F.); 2 Department of Physics of Condensed Matter, Optics Area, Pharmacy Faculty, University of Seville, 41012 Seville, Spain

**Keywords:** dry eye disease, tear film, ocular surface, agricultural workers, environmental exposure, Colombia

## Abstract

**Background/Objectives:** This study aimed to evaluate the relationship between cumulative occupational exposure and ocular surface alterations in Colombian tomato farm workers, using data collected through a cross-sectional survey. In addition, the study sought to explore how occupational exposure duration may support risk stratification and targeted preventive strategies in this vulnerable population. **Methods:** A cross-sectional observational study was conducted involving 72 tomato farm workers in Colombia. Participants were grouped according to duration of agricultural work experience (<15 years vs. ≥15 years). Clinical assessments included slit lamp examination, tear film break-up time (BUT), Schirmer test, and fluorescein staining. Subjective symptoms were evaluated using the McMonnies Dry Eye Questionnaire. Ocular surface alterations, including conjunctival changes and Meibomian gland dysfunction, were documented and statistically analyzed between groups. **Results:** Workers with ≥15 years of experience reported significantly higher dry eye symptom scores (McMonnies mean = 8.19 ± 2.54) than those with <15 years (mean = 6.59 ± 2.61; *p* = 0.006). Schirmer test scores were lower in the experienced group (16.30 ± 11.48 mm vs. 22.71 ± 11.20 mm; *p* = 0.018), indicating reduced tear production. Bulbar conjunctival alterations and Meibomian gland obstruction were significantly more frequent in the experienced group (*p* = 0.002 and *p* = 0.013, respectively). No significant differences were found in BUT or eyelid findings. **Conclusions:** Long-term agricultural work was associated with increased dry eye-related symptoms and clinical signs of ocular surface compromise among Colombian tomato farm workers. From a personalized medicine perspective, occupational exposure duration may represent a useful risk-stratification factor to identify workers who could benefit from targeted screening, preventive counseling, protective interventions, and individualized follow-up. These findings support the implementation of tailored occupational eye health strategies to reduce cumulative ocular surface damage in vulnerable rural populations.

## 1. Introduction

Agriculture remains a fundamental pillar of rural identity and economic subsistence in Colombia. Tomato farming, especially in open-field systems, provides income for thousands of families and forms part of the country’s diversified horticultural economy. In Colombia, agricultural workers are exposed to specific environmental conditions, including high levels of ultraviolet radiation, dust, and agrochemical exposure, which may contribute to ocular surface alterations [1]. Despite this, data on dry eye disease and ocular surface health in this population remain limited [2,3]. Smallholder farming and labor-intensive practices remain common in Colombia and resemble conditions reported in other developing countries, where occupational injury prevention programs are often limited [4]. Consistent with global intensification of agriculture, Colombia has witnessed an increase in pesticide use [5], following a trend similar to that in other developing nations [6]. While agrochemicals have supported productivity, their misuse—such as exceeding concentrations and poor application practices—has been linked to systemic health conditions and localized ocular toxicity [7]. Farmers are often unaware of the cumulative risks, and national surveillance of pesticide-related morbidity remains scarce. Chronic exposure through spraying and environmental contact raises urgent concerns for rural health and highlights the need for occupational risk profiling to identify workers who may benefit from targeted preventive strategies and individualized ocular health surveillance [8].

Agricultural labor exposes workers to a variety of physical and chemical hazards. Prolonged exposure to ultraviolet radiation, wind, particulate matter, and aerosols increases the risk of ocular damage. These stressors act through skin, respiratory epithelium, and ocular mucosa, often without protective barriers in place [8,9,10]. Exposure to high temperatures and airborne contaminants, as observed in biomass-burning or harvesting conditions, has been associated with increased ocular surface inflammation and epithelial dysfunction [11]. These risks are exacerbated by long hours, lack of rest periods, and inadequate regulation of occupational environments [12].

The eye’s surface is especially vulnerable to environmental and chemical insults. Dry eye disease (DED)-related symptoms are frequently reported among exposed populations [10,12,13]. Increased apoptosis and impaired cellular regeneration associated with chronic toxic exposure have also been described [7]. Studies in tropical agricultural settings confirm that cumulative exposure to pollutants, low humidity, and poor air quality can significantly affect tear stability and conjunctival health [4,11].

Despite global recommendations, the use of personal protective equipment (PPE) among agricultural workers remains low [4,6]. Factors such as discomfort, lack of availability, and sociocultural attitudes hinder PPE adoption. The resulting gap in protection increases the likelihood of chronic ocular surface damage and trauma [14,15]. Long-term agricultural exposure may be associated with a measurable decline in ocular surface integrity; however, the relationship between cumulative exposure duration and ocular surface alterations remains insufficiently characterized. As seen in other agricultural communities, underestimation of risk leads to underreporting and insufficient policy response [14,15]. The municipality of Suratá, located in the north of the Santander department, represents a relevant rural setting for studying occupational ocular risk. It covers an area of 734.48 km^2^, with temperatures ranging from 13 °C to 19 °C. Its main economic activities include mining, agriculture, and cattle raising.

The diagnosis of dry eye disease (DED) is complex and typically requires a combination of subjective and objective assessments. Commonly used clinical tests include tear film break-up time, Schirmer test, and ocular surface staining, which evaluate tear stability, aqueous production, and epithelial integrity, respectively. In parallel, validated questionnaires such as the McMonnies Dry Eye Questionnaire are widely used to assess symptom severity [16,17,18]. However, it is well recognized that signs and symptoms of DED often show poor correlation, and no single test is sufficient to establish a definitive diagnosis. Therefore, a multifactorial diagnostic approach is recommended in both clinical and research settings [19]. In occupational populations, this multidimensional assessment may also support individualized risk stratification by identifying workers with higher symptom burden, reduced tear production, tear film instability, or ocular surface damage who may benefit from targeted preventive counseling, protective measures, and tailored clinical follow-up.

Therefore, the aim of this study was to evaluate the relationship between cumulative agricultural exposure and ocular surface changes among Colombian tomato farm workers, using data collected in a cross-sectional setting. In line with a personalized medicine approach, the study also sought to determine whether exposure duration may help identify workers at greater ocular surface risk and guide targeted occupational eye health strategies.

## 2. Materials and Methods

### 2.1. Study Design and Setting

This research was conducted as a cross-sectional study with prospective data collection to investigate ocular surface alterations in tomato farm workers. The design involved a structured sampling framework and standardized data collection procedures to ensure reliability and reproducibility. Sample size was calculated using the standard formula for prevalence studies [19], *n* = Z^2^ *p* (1 − *p*)/d^2^, assuming an expected prevalence of 65% [20], a 90% confidence level, and 10% precision. The initial estimated sample size was 62 participants; after applying a 5% allowance for possible non-response or incomplete data, the minimum required sample was 65 participants. Data were prospectively collected between 9 June 2024 and 18 June 2024. The study was coordinated by researchers from the Faculty of Optometry at Universidad Santo Tomás, Bucaramanga, and the fieldwork was conducted in rural areas of the municipality of Suratá, Santander, Colombia. Surata is located within the same Colombian department/region where the Bucaramanga campus is based and falls within the community-based research activities of the institution. The fieldwork site and data source were therefore included within the institutional scope and ethical oversight of Universidad Santo Tomás, Bucaramanga. Sampling followed a stratified random procedure. Initially, a census of agricultural activity was conducted across 22 geographic subdivisions within the municipal boundaries. Of these, nine regions with accessible road infrastructure were selected for fieldwork. Farms engaged specifically in tomato cultivation were identified within these areas, and eligible participants were recruited accordingly. The study was conducted in the municipality of Suratá, located in the Santander department in northeastern Colombia. Participants were recruited from multiple rural veredas within the municipality, including República, Llano Grande, Laguna, San Lorenzo, Pueblo Nuevo, Chumbula, Carrisal, Porvenir, Cartagua, and Villa. These areas represent typical smallholder agricultural settings where tomato farming is conducted under both open-field and greenhouse conditions. This distribution ensured a geographically diverse representation of the farming population within the study area. This recruitment strategy also allowed the study to capture workers exposed to heterogeneous occupational and environmental conditions, which is relevant for identifying exposure profiles that may inform individualized ocular health surveillance. The study was conducted in accordance with the principles outlined in the Declaration of Helsinki. All participants provided informed consent prior to inclusion. The study protocol was reviewed and approved by the Ethics Committee of Saint Thomas University (code 0189-2024 and date: 12 February 2024).

### 2.2. Study Population and Eligibility Criteria

The study population consisted of individuals actively engaged in tomato farming within selected rural zones of the municipality of Suratá (Santander, Colombia), including multiple rural veredas identified through a prior agricultural census. Eligible participants were adults aged 18 years or older who had been continuously involved in tomato cultivation for a minimum period of six months. These criteria were established to ensure sufficient occupational exposure relevant to the study objectives. Participants were stratified according to years of agricultural exposure (<15 years vs. ≥15 years). This threshold was selected as an approximation of cumulative occupational exposure and was supported by the distribution of exposure years within the study sample, which showed a natural separation around this value. This approach allowed differentiation between lower and higher exposure groups for comparative analysis in the absence of standardized exposure thresholds in the literature. From a personalized occupational health perspective, this stratification also allowed exploration of whether exposure duration could serve as a practical risk-stratification variable for identifying workers who may require closer ocular surface monitoring. Participants were recruited from farms identified through a prior census and stratified random sampling process, as described in the study design. All individuals were approached in person and informed about the purpose and procedures of the study. Those who voluntarily agreed to participate provided written informed consent before undergoing any evaluation. To maintain the clinical consistency of ocular surface assessments and minimize confounding factors, specific exclusion criteria were applied. Individuals were excluded if they had a history of ocular refractive surgery, which could independently affect tear film or corneal parameters. Pregnant women were also excluded due to potential physiological changes that could influence ocular surface conditions.

### 2.3. Clinical Assessment and Outcome Measurements

The study evaluated a comprehensive set of variables across three primary domains: demographic and work characteristics, ocular symptoms and history, and detailed ocular examination findings. Demographic and occupational data included participants’ age and sex, as well as their highest level of education attained. Agricultural work profiles were characterized by the type of cultivation practiced (greenhouse, open field, or both), the number of years of agricultural experience, average daily working hours, and the number of working days per week. The use of personal protective equipment, specifically protective glasses, was recorded. Participants also reported engagement in specific agricultural tasks, including fumigation, harvesting, planting, and soil preparation. The type of agrochemical exposure was categorized based on the use of pesticides, fertilizers, or both. Self-reported history of arthritis and thyroid disorders was also captured, as these systemic conditions have been associated with ocular surface alterations and dry eye disease in the previous literature [21,22]. These occupational and clinical variables were selected to characterize individual exposure profiles and to explore factors potentially relevant to personalized risk assessment in agricultural workers.

Ocular history and symptomatology encompassed the presence of symptoms such as dryness, burning, and itching. The use of treatments for dry eye was documented. Participants reported the frequency of mucosal dryness and sensitivity to environmental irritants such as smoke or air, as well as whether they typically slept with their eyes open and experienced discomfort upon waking. Additional information was obtained regarding contact lens use. Additionally, the McMonnies Dry Eye Questionnaire was administered to all participants, and the total score was calculated to quantify the severity of dry eye symptoms [18,23,24]. Clinical ocular examinations provided objective assessments of ocular surface health. Observations included the external appearance of the eyelids and eyelashes and the condition of the bulbar and tarsal conjunctiva. Corneal transparency and iris coloration were recorded, along with lens status. Tear film function was assessed through measurements of tear meniscus height, evaluation of meibomian gland secretion, and analysis of corneal staining patterns. Quantitative measures included tear break-up time (in seconds) [1,25,26] and Schirmer test results (in millimeters) [27,28], both of which are standard indicators of tear film stability and aqueous tear production, respectively.

For the interpretation of clinical parameters, tear film break-up time values below 10 s were considered indicative of tear film instability, and Schirmer test values below 10 mm were considered suggestive of reduced aqueous tear production. Ocular surface staining was qualitatively assessed as an indicator of epithelial compromise. It should be noted that, in accordance with current consensus reports, the diagnosis of dry eye disease requires the presence of both symptoms and at least one positive clinical sign [16]. In the present study, the objective was to evaluate ocular surface alterations and dry eye-related parameters; therefore, both subjective symptoms and objective clinical findings were analyzed, but a formal diagnosis of dry eye disease was not established. This approach allowed assessment of clinically relevant ocular surface patterns without overclassifying participants as having formally diagnosed dry eye disease.

### 2.4. Statistical Analysis

All statistical analyses were performed using IBM SPSS Statistics version 29 (IBM Corp., Armonk, NY, USA). The normality of quantitative variables was assessed using the Kolmogorov–Smirnov test. Descriptive statistics were calculated for all variables. Categorical variables were summarized as frequencies and percentages, while continuous variables were expressed as mean ± standard deviation (SD) or median and interquartile range (IQR), depending on their distribution. Participants were stratified into two groups based on agricultural experience: those with <15 years and those with ≥15 years of experience. Group comparisons were performed to assess the influence of agricultural experience on the variables of interest. For qualitative (categorical) variables, the Chi-square test or Fisher’s exact test (when expected cell counts were <5) was used. For quantitative variables, the Mann–Whitney U test was applied, as most data did not follow a normal distribution. A two-tailed *p*-value of less than 0.05 was considered statistically significant. The 95% confidence intervals (CIs) were reported where applicable. No imputation was made for missing data. Data cleaning and validation steps were undertaken before analysis to ensure data integrity.

## 3. Results

### 3.1. Demographic and Occupational Characteristics

A total of 72 Colombian tomato farm workers were included in the study. Demographic and occupational variables included age, sex, education level, rural work area, cultivation type, agricultural work experience, working hours per day, working days per week, use of protective glasses, agricultural tasks performed, type of agrochemical exposure, and self-reported history of arthritis and thyroid disease. These characteristics are summarized in Table 1.

### 3.2. Ocular Symptoms and Subjective Findings

Variables related to ocular symptoms and subjective findings included dryness, burning, itching, use of dry eye treatment, sensitivity to smoke or air, mucosal dryness, sleeping with eyes open, morning ocular discomfort, contact lens use, and McMonnies Dry Eye Questionnaire score. These variables are presented in Table 2.

### 3.3. Clinical Ocular Surface Findings

Clinical ocular surface variables included eyelid and eyelash findings, bulbar and tarsal conjunctival abnormalities, corneal transparency, iris appearance, lens integrity, tear meniscus height, Meibomian gland function, tear film break-up time (BUT), fluorescein staining, and Schirmer test results. These findings are presented in Table 3.

### 3.4. Comparison by Agricultural Exposure Duration

Based on the duration of agricultural work experience, participants were categorized into two groups: those with less than 15 years (*n* = 41) and those with 15 years or more of experience (*n* = 31). Comparative analyses were then performed to evaluate differences in ocular symptoms and ocular surface clinical parameters between these two groups. The findings from the ocular examination and tear evaluation are summarized below. This comparison was intended to explore whether cumulative occupational exposure could identify workers with a higher burden of ocular surface alterations and therefore support targeted screening and individualized preventive strategies.

### 3.5. Eyelid and Eyelash Status

No significant differences were found in eyelid status between the two groups. Most participants showed normal coloration, with minor abnormalities such as ptosis or irregularity appearing only in two individuals (Chi-square = 2.080, *p* = 0.353). Similarly, eyelash appearance was normal, with collarettes observed only in Group 2 (Chi-square = 2.080, *p* = 0.353).

### 3.6. Corneal, Iris and Lens Findings

Corneal transparency was preserved in all eyes, with one mild opacity recorded in Group 2. No significant difference was found (Chi-square = 1.341, *p* = 0.247; Fisher’s exact *p* = 0.431). Iris color was mostly brown across both groups. Other findings (blue/green iris or pigmentation) were rare and not significantly different (Chi-square = 4.822, *p* = 0.185). The crystalline lens was intact in all but one case in Group 2, which showed opacity. No statistically significant difference was observed (Chi-square = 1.141, *p* = 0.247; Fisher’s exact *p* = 0.431).

### 3.7. Tear Meniscus and Conjunctival Findings

Tear meniscus height was intact (82.9% in Group 1 vs. 74.1% in Group 2), with no significant difference (Chi-square = 0.952, *p* = 0.621). A significant difference was found in the bulbar conjunctiva: 70.7% of Group 1 showed normal appearance vs. 32.2% in Group 2, with higher alteration rates in the experienced group (Chi-square = 10.525, *p* = 0.001; Fisher’s exact *p* = 0.002). In contrast, tarsal conjunctiva classification did not differ significantly (Chi-square = 0.062, *p* = 0.803). These findings suggest that longer occupational exposure may be associated with more frequent conjunctival alterations, which could help identify workers requiring closer ocular surface surveillance.

### 3.8. Meibomian Gland Secretion and Fluorescein Staining

Obstructed Meibomian glands were significantly more frequent in Group 2 (54.8%) than in Group 1 (24.3%), indicating a statistically significant difference (Chi-square = 6.983, *p* = 0.008; Fisher’s exact *p* = 0.013). Negative staining predominated in both groups. Linear staining was more frequent in Group 2 (16.16% vs. 4.8%), but the difference did not reach statistical significance (Chi-square = 2.720, *p* = 0.257).

### 3.9. Subjective Symptoms, Tear Film Stability and Aqueous Production

**Figure 1:**
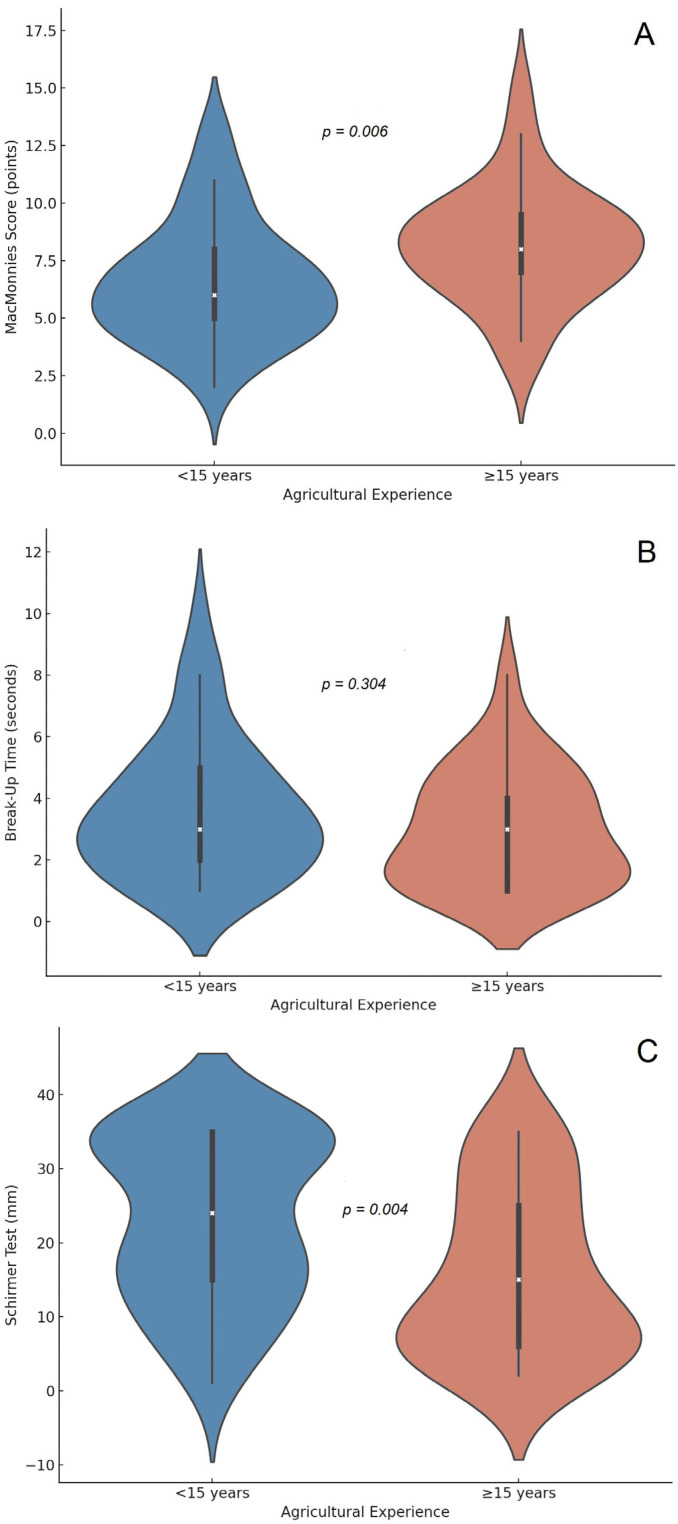
Ocular surface parameters according to agricultural experience in tomato farmers from Colombia. (**A**) McMonnies questionnaire score (points), (**B**) tear film break-up time (seconds), and (**C**) Schirmer test (millimeters) were compared between farmers with less than 15 years and those with 15 years or more of agricultural experience. Violin plots display the distribution, median, and interquartile range of each variable. The corresponding *p*-values were calculated using the Mann–Whitney U test.

A statistically significant difference was found in subjective ocular symptomatology as measured by the MacMonnies questionnaire. Participants with ≥15 years of experience reported higher symptom scores (mean = 8.19 ± 2.54) compared to those with <15 years (mean = 6.59 ± 2.61). This difference was confirmed by the Mann–Whitney U test (U = 393.5, Z = −2.772, *p* = 0.006), indicating greater ocular discomfort among the more experienced group (Figure 1A). BUT values were similar between groups (3.42 ± 2.04 s vs. 3.30 ± 2.19 s; Mann–Whitney U = 591.5, *p* = 0.678) (Figure 1B). However, the Schirmer test revealed a statistically significant reduction in tear production among the more experienced professionals (22.71 ± 11.20 mm in Group 1 vs. 16.30 ± 11.48 mm in Group 2; Mann–Whitney U = 437.5, *p* = 0.018) (Figure 1C).

## 4. Discussion

This study evaluated ocular surface alterations and dry eye-related symptoms among 72 Colombian tomato farm workers, comparing individuals with less than 15 years of agricultural work experience with those with 15 years or more. While no significant differences were observed in eyelid, eyelash, corneal, iris, or lens findings, significant alterations were noted in parameters associated with chronic ocular surface stress. Workers with longer exposure demonstrated significantly more bulbar conjunctival abnormalities and obstructed Meibomian glands. Moreover, these individuals reported greater ocular discomfort, as reflected in higher MacMonnies symptom scores, and exhibited reduced aqueous tear production measured by the Schirmer test. Although tear film break-up time (BUT) did not significantly differ between groups, the data suggest a progressive decline in ocular surface health with prolonged exposure to agricultural conditions, particularly regarding tear volume and Meibomian gland function. From a personalized occupational health perspective, these findings suggest that duration of agricultural exposure may help identify workers who require targeted screening, preventive counseling, reinforcement of protective measures, and individualized ocular surface follow-up.

An additional relevant finding is the consistently low tear break-up time observed across both exposure groups, indicating a generalized tear film instability in the study population. This suggests that even individuals with shorter exposure duration may already experience significant ocular surface compromise, likely due to continuous environmental and occupational stressors. As a result, the comparison between groups may underestimate the overall impact of agricultural exposure, as both groups appear to be affected beyond normal physiological values. This supports the notion of a high baseline burden of ocular surface dysfunction in this population. Therefore, preventive strategies should not be restricted only to workers with longer exposure duration but should also consider early screening in newly or moderately exposed workers.

The absence of significant differences in tear break-up time despite reductions in Schirmer test values and increased Meibomian gland dysfunction suggests a complex and potentially mixed pattern of ocular surface involvement. While reduced tear production points toward an aqueous-deficient component, the presence of Meibomian gland obstruction supports an evaporative mechanism. The consistently low BUT values observed across both groups may indicate a baseline level of tear film instability affecting the entire population, likely related to environmental exposure. Together, these findings suggest that occupational conditions may induce a mixed or nonspecific ocular surface stress response rather than a single dry eye subtype.

Overall, these findings indicate a pattern of ocular surface compromise associated with cumulative occupational exposure, consistent with previously reported environmental risk models, while also highlighting specific alterations in tear production and Meibomian gland function. This combined clinical profile may be useful for developing personalized occupational eye health strategies based on exposure duration, symptom burden, and objective ocular surface findings.

The ocular surface alterations observed in this study—particularly bulbar conjunctival abnormalities, Meibomian gland dysfunction, and reduced tear production among long-term agricultural workers—are consistent with findings reported in similar environmental and occupational settings. In a large population-based study in India, Tandon et al. [12] found that dry eye disease (DED) was more prevalent among people engaged in outdoor work and in regions with higher sunlight exposure. Notably, exposure to sunlight (OR = 1.8), smoke, and lower humidity were significant risk factors for DED. These findings are consistent with previous reports; however, the present study specifically demonstrates that cumulative exposure duration may play a more relevant role than occupational category alone. This distinction is important from a personalized medicine perspective, because exposure duration may help refine individual risk profiles beyond broad occupational classification.

Febriana et al. [6], in a study of Indonesian vegetable farmers, reported a 28.78% prevalence of dry eye syndrome based on the Schirmer test and OSDI, similar to the clinical patterns we observed. Their population also had poor PPE use and extensive pesticide exposure, which has been associated with oxidative stress on the ocular surface. Likewise, Sanyal and Law [7] mechanistically demonstrated that chronic pesticide exposure induced apoptosis and reduced cellular proliferation in corneal epithelial cells in a murine model. This biological evidence supports the hypothesis that long-term agrochemical exposure contributes not only to functional impairment of the tear film but also to structural damage of the ocular surface over time, as likely seen in our high-exposure group. These findings support a potential mechanism in which chronic environmental exposure contributes to both inflammatory and functional alterations of the ocular surface.

Matsuda et al. [11] further showed that workers exposed to sugarcane biomass burning had altered mucin gene expression (increased MUC16, reduced neutral mucus), which mirrors the conjunctival abnormalities we documented. These findings indicate that mucosal changes from chronic environmental exposure are not only symptomatic but also underpinned by biochemical and histological alterations. Similar mechanisms may be implicated in the tomato farmers we evaluated, particularly in those with bulbar conjunctival changes and reduced tear meniscus.

The relationship between occupation and dry eye was also emphasized by Lee et al. [13] and Bazeer et al. [9], who found that indoor and screen-intensive professions had higher DES risk. However, Bazeer et al. [9] paradoxically noted a lower risk in outdoor occupations such as farming. This contrasts with our results and those from India and Indonesia, suggesting that geographic factors, agricultural intensity, or lack of protective behaviors (like PPE use) may account for regional differences in risk. These discrepancies further support the need for context-specific risk assessment rather than assuming uniform occupational risk across all agricultural settings.

Lastly, Liou et al. [10] linked dry eye metrics to metal biomarker levels in welders, showing that chronic exposure to airborne pollutants, regardless of industry, can alter tear physiology. This reinforces the notion that occupational air quality, not just chemical class, is a pivotal driver in DED.

### Limitation and Future Research Lines

This study offers important insights into ocular effects of long-term agricultural exposure, but several limitations should be noted. Its cross-sectional design prevents causal inference, although the clear differences between experience groups suggest a cumulative effect. Advanced diagnostics (e.g., tear osmolarity, ocular surface imaging) were not used, limiting our ability to differentiate dry eye subtypes. Exposure data were self-reported and may suffer from recall bias. Additionally, we were unable to explore variations in the level of risk associated with this exposure due to the lack of accurate data on the agrochemicals used. Although the McMonnies questionnaire is a widely used and standardized tool for the assessment of dry eye symptoms, its application across different populations may be influenced by environmental and cultural factors, which could affect symptom reporting.

Basic ophthalmic parameters such as uncorrected and best corrected visual acuity were not included in the assessment, which may limit the clinical characterization of the study population. Although general categories of agrochemical exposure and agricultural tasks were recorded, the study was not designed to perform stratified analyses according to specific pesticide types, application practices, or cultivation modalities, which may have differential effects on ocular surface health. This limitation is particularly relevant, as different pesticide classes, spraying practices, and cultivation conditions may have distinct effects on ocular surface health and dry eye disease risk. Future studies should incorporate more detailed exposure characterization to better understand these associations.

Future studies should include objective environmental or biological exposure measurements, as shown in [10], and explore worker attitudes toward PPE through qualitative methods. Our sample size, while sufficient for group comparisons, limits stratified analyses. Broader, multicenter studies across Latin America are needed to validate and expand these findings.

This study focused on ocular surface parameters, but other pathologies like cataract or retinal changes may also be relevant in this population. Future research should adopt longitudinal designs, apply comprehensive ophthalmic assessments, and assess preventive interventions such as safety eyewear and lubricants.

From a personalized medicine perspective, the findings of this study highlight the importance of occupational exposure duration, work profile, and environmental conditions as individual risk factors contributing to variability in ocular surface health. These parameters may enable risk stratification within agricultural populations, facilitating targeted screening, early detection, and preventive strategies for individuals at higher risk of ocular surface alterations. Such an approach is particularly relevant in underserved rural settings, where tailored interventions may improve long-term ocular health outcomes. Future studies should also evaluate whether personalized occupational eye health protocols based on exposure duration, symptom burden, PPE adherence, and objective ocular surface findings can reduce the incidence or progression of ocular surface alterations in agricultural workers.

## 5. Conclusions

Prolonged occupational exposure in tomato farming is associated with significant ocular surface alterations, including increased conjunctival changes, Meibomian gland dysfunction, and reduced tear production. Workers with 15 or more years of experience reported greater ocular discomfort, highlighting the cumulative impact of agricultural work on ocular health. These findings support the need for targeted preventive strategies, including regular ocular assessments, consistent use of protective eyewear, and occupational health education in agricultural settings. From a personalized medicine perspective, exposure duration, work profile, and individual ocular surface findings may help identify workers who would benefit from closer monitoring, tailored preventive counseling, and individualized ocular surface management.

## Figures and Tables

**Table 1:** Demographic characteristics.

Demographics	
Age (years), mean ± SD	39.5 (27)
Sex, male (%)	51 (70.8)
Education level	
None	8 (11.1)
Primary	29 (40.3)
Technical	4 (5.6)
High School	31 (43.1)
Labor Farm	
República	21 (29.2)
Laguna	10 (13.9)
San Lorenzo	10 (13.9)
Pueblo Nuevo	9 (12.5)
Llano Grande	7 (9.7)
Chumbula	5 (6.9)
Porvenir	4 (5.6)
Carrisal	3 (4.2)
Villa	2 (2.8)
Cartagua	1 (1.4)
Cultivation Type	
Greenhouse	19 (26.4)
Open field	41 (56.9)
Both	12 (16.7)
Work conditions	
Agriculture experience, years	3 (24.8)
Working hours/day	8 (0)
Working days/week	6 (1)
Use of Glasses as PPE, yes (*n*, %)	20 (27.8)
Agriculture Task	
Fumigations, (*n*, %)	46 (63.9)
Harvesting, (*n*, %)	63 (87.5)
Planting, (*n*, %)	57 (79.2)
Soil preparation (*n*, %)	45 (62.5)
Agrochemical Use	
Pesticides, *n* (%)	10 (14.1)
Fertilizers, *n* (%)	14 (19.7)
Both, *n* (%)	47 (66.2)
Arthritis Status	
Yes, *n* (%)	1 (1.4)
No, *n* (%)	60 (84.5)
Not known, *n* (%)	10 (14.1)
Thyroid Disorder	
Yes, *n* (%)	0 (0)
No, *n* (%)	64 (0)
Not known, *n* (%)	6 (8.6)
PPE: Personal Protective Equipment	

**Table 2:** Ocular symptoms, history, and subjective characteristics.

Ocular Symptoms (Multiple Responses)	
Dryness, *n* (%)	33 (45.8)
Burning, *n* (%)	39 (54.2)
Itching, *n* (%)	22 (30.6)
Dry Eye Treatment	
Yes, *n* (%)	25 (34.7)
No, *n* (%)	47 (65.3)
Mucosal Dryness Frequency	
Never, *n* (%)	50 (69.4)
Sometimes, *n* (%)	19 (26.4)
Often, (%)	3 (4.2)
Smoke or Air Sensitivity	
Never, *n* (%)	24 (33.3)
Sometimes, *n* (%)	17 (23.6)
Often, *n* (%)	31 (43.1)
Sleep with Open Eyes	
Never, *n* (%)	40 (55.6)
Sometimes, (*n*, %)	1 (1.4)
Often, (*n*, %)	5 (6.9)
Not known, (*n*, %)	26 (36.1)
Morning Discomfort	
Never, *n* (%)	41 (56.9)
Sometimes, *n* (%)	19 (26.4)
Often, *n* (%)	12 (16.7)
Contact Lens User	
No, *n* (%)	71 (98.6)
Yes, *n* (%)	1 (1.4)
MacMonnies Questionnaire (Score Points)	7 (4)

**Table 3:** Ocular characteristics.

Eyelid Status	Right Eye	Left Eye
Normal coloration, *n* (%)	70 (97.2)	70 (97.2)
Ptosis, *n* (%)	1 (1.4)	1 (1.4)
Irregular eyelid, *n* (%)	1 (1.4)	1 (1.4)
Eyelash Status		
Normal, *n* (%)	70 (97.2)	70 (97.2)
Collarettes, *n* (%)	1 (1.4)	1 (1.4)
Crusts, *n* (%)	1 (1.4)	1 (1.4)
Bulbar Conjunctiva		
Smooth, *n* (%)	40 (55.6)	40 (55.6)
Pterigium, *n* (%)	13 (18.1)	11 (15.3)
Pinguecula, *n* (%)	9 (12.5)	17 (23.6)
Striae, *n* (%)	4 (5.6)	2 (2.8)
Hyperpigmentation, *n* (%)	2 (1.4)	0 (0)
Generalized hyperemia, *n* (%)	2 (2.8)	1 (1.4)
Filamentous secretion, *n* (%)	2 (2.8)	1 (1.4)
Tarsal Conjunctiva		
Smooth, *n* (%)	43 (33.3)	44 (66.1)
Papillary reaction, *n* (%)	27 (37.5)	26 (36.1)
Generalized hyperemia, *n* (%)	3 (4.2)	3 (4.2)
Concretions, *n* (%)	1 (1.4)	1 (1.4)
Cornea Transparency		
Clear, *n* (%)	70 (97.2)	71 (98.6)
Mild opacity, (*n*, %)	2 (2.8)	1 (1.4)
Iris Color		
Brown, *n* (%)	68 (94.4)	69 (95.8)
Green, *n* (%)	2 (2.8)	2 (2.8)
Blue, *n* (%)	1 (1.4)	1 (1.4)
Unclear, *n* (%)	1 (1.4)	1 (1.4)
Lens Status		
Complete and in place, *n* (%)	71 (98.6)	71 (98.6)
Opacities present, *n* (%)	1 (1.4)	0 (0)
Not assessable, *n* (%)	0 (0)	1 (1.4)
Tear Meniscus Height		
Decreased, *n* (%)	11 (14.9)	11 (14.9)
Intact (normal), *n* (%)	58 (78.4)	58 (78.4)
Increased, *n* (%)	5 (6.8)	5 (6.8)
Meibomian Glands		
Normal secretion, *n* (%)	50 (67.6)	50 (67.6)
Obstructed, *n* (%)	24 (32.4)	24 (32.4)
Corneal staining		
Negative, *n* (%)	55 (77.5)	64 (90.1)
Linear, *n* (%)	11 (15.5)	4 (5.6)
Punctate, *n* (%)	5 (7.0)	3 (4.2)
Tear Break-Up Time (s)	3 (3)	3 (4)
Schirmer Test (mm)	18.5 (26)	23 (25)

## Data Availability

The data presented in this study are available on request from the corresponding author due to research project embargo reasons.

## Abbreviations

The following abbreviations are used in this manuscript:

BUT	Tear Break-Up Time
CI	Confidence Interval
DED	Dry Eye Disease
IQR	Interquartile Range
OR	Odds Ratio
PPE	Personal Protective Equipment
